# Mapping of Children’s Palliative Care Development Globally in 2023

**DOI:** 10.3390/children12040440

**Published:** 2025-03-30

**Authors:** Julia Downing, Lizzie Chambers, Alex Daniels, Julie Ling, Ednin Hamzah, Emmanuel Luyirika, Regina Okhuysen-Cawley, Megan Doherty, Justin N. Baker

**Affiliations:** 1 International Children’s Palliative Care Network, Bristol BS1 2NT, UK; programme.manager@icpcn.org (L.C.); education@icpcn.org (A.D.); rxokhuys@texaschildrens.org (R.O.-C.); jusbaker@stanford.edu (J.N.B.); 2 International Children’s Palliative Care Network, Durban 3624, South Africa; 3 European Association for Palliative Care, 1800 Vilvoorde, Belgium; lingp@who.int; 4 Asia Pacific Hospice Palliative Care Network, Singapore 168583, Singapore; ednin@hospismalaysia.org; 5 African Palliative Care Association, Kampala P.O. Box 72518, Uganda; emmanuel.luyirika@africanpalliativecare.org; 6 Latin American Palliative Care Association, Bogota 110221, Colombia; 7 Two Worlds Cancer Collaboration, North Vancouver, BC V7H 2Y8, Canada; megandoherty@hey.com; 8 Division of Quality of Life and Pediatric Palliative Care, Stanford Medicine Children’s Health and Stanford University, Palo Alto, CA 94305, USA

**Keywords:** children, paediatric/pediatric, palliative care, global, mapping

## Abstract

**Background:** The demand for children’s palliative care is increasingly urgent, with over 21 million children worldwide needing access, yet only 5–10% currently receiving it. Mapping the development of children’s palliative care is important in tracking progress and focusing priorities for future work. **Methods:** In 2023, a cross-sectional survey was conducted to assess the status of children’s palliative care globally, focusing on eight key indicators. Children’s palliative care experts and stakeholders were asked to evaluate the perceived levels of development in their countries across five defined levels, ranging from no known provision to broad integration into health care services. Efforts were made to engage non-responding countries. Regional palliative care associations were consulted to validate the results. **Results:** The survey was distributed to experts in 167/197 countries (85%), achieving data collection for 131 countries (78% of surveys sent). A total of 42% of countries (83) were at the lowest level of development (Level 1), while only 6% (11) of countries reached the highest level (Level 5), showing significant regional variation. An overall increase in children’s palliative care globally was seen, including significant movement between levels. Variations were seen between regions and across country income categories, providing insight to inform ongoing efforts in service development, advocacy, education, research and access to essential medicines. **Conclusions:** There has been global progress in children’s palliative care, although substantial gaps remain in access, particularly between high-income countries and low- and middle-income countries (LMICs). The study highlights the importance of a comprehensive approach to children’s palliative care, with advocacy and education and training programmes being crucial for sustainable development.

## 1. Introduction

There are over 21 million children globally requiring access to palliative care [1], and with this number expected to increase, the need for children’s palliative care has never been greater. Yet it is estimated that only 5–10% of children and their families needing palliative care around the world can access it [2,3]. Since its inception in 2005 the International Children’s Palliative Care Network has held a directory of services and has mapped the provision of children’s palliative care at the country level. The latest mapping undertaken in 2023 aimed to further understand the state of children’s palliative care provision globally, how it has developed over the past twenty years, the key barriers and challenges facing children’s palliative care development and its integration into Universal Health Coverage (UHC) (as recommended by UHC 2030—WHO 2025) [4]. This information is vital to inform and underpin future activities to support service development, advocacy, education, capacity building, research, and access to essential medicines. This paper aims to discuss the 2023 mapping, highlighting key changes and developments.

## 2. Children’s Palliative Care Development

**Figure 1:**
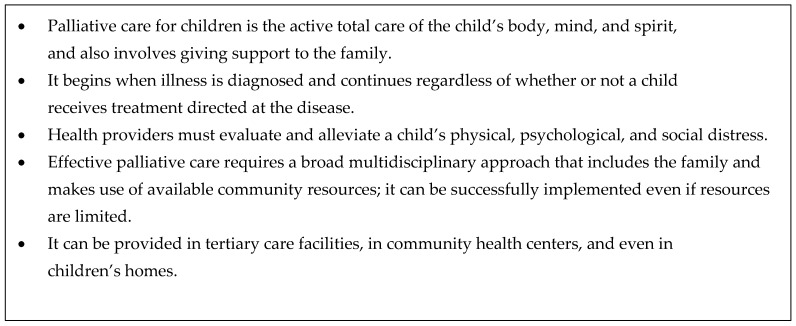
World Health Organization (WHO) definition of palliative care for children (WHO 2023).

Children’s palliative care is a separate, albeit closely related, field to palliative care for adults and was first defined by the World Health Organisation (WHO) in 1998 [5]. Palliative care for children is “the active total care of the child’s body, mind and spirit and also involves giving support to the family” [6] and should be provided for all children with a life-limiting or life-threatening condition (Figure 1). Whilst palliative care for adults is well established, in many countries the development of children’s palliative care has lagged behind that of adults [7], and challenges continue for the ongoing development of services [8].

In 2011, Knapp et al. published a systematic review that looked at the provision of children’s palliative care around the world in response to a dearth of information on progress to development [9]. They reviewed 117 papers categorising the development according to four levels of palliative care development. These four categories (no known activity, capacity building, localised provision and approaching integration) were identified by Wright et al. in one of the first papers mapping palliative care development globally [10]. Based on this categorisation, they reported that 65.6% of countries had no known activity on children’s palliative care, 18.8% had capacity-building activities, ~10% had localised provision, and only 5.7% of countries had a provision that was reaching mainstream providers. This review identified large gaps and showed the disparities between regions, with 85.7% of countries in the Oceanic region being at level 1, 81.1% of African countries, 78.3% of North American countries, 66.7% of South American countries, 65.9% of Asian countries and only 32.6% of countries in Europe. The Oceanic region also had a very skewed distribution, with 85.7% countries at level 1, i.e., no known activity and 14.3% at level 4, i.e., approaching integration, with no countries in the middle [9].

Since the initial systematic review by Knapp et al. [9] and the foundational work by Wright et al. [10], further efforts have been made to map global palliative care delivery. The Global Atlas of Palliative Care at the End of Life [11] built on Knapp et al.’s [9] identification of children’s palliative care development levels and highlighted that most services were concentrated in high-income countries. This edition of the Atlas underscored the importance of tracking the development of children’s palliative care services worldwide to assess each country’s progress, identify necessary support, and guide advocacy efforts. Additionally, it recognised the need to expand Wright et al.’s [10] original four categories to capture a more nuanced view of development, reflecting the evolving nature of children’s palliative care and its impact on the tracking process.

Further developments with regard to the categories used, the methods for estimating country levels and developments, the publication of global indicators for palliative care development [12] and regional atlases [13,14,15,16,17] have all contributed significantly to the global mapping of palliative care, and therefore children’s palliative care. The regional atlases for Latin America [13,17], Africa [14], the Eastern Mediterranean Region [15] and Europe [16] have been significant in the development of global indicators and in the evolving methodology for mapping the development of palliative care for children. The regional atlases utilised the WHO Public Health framework for palliative care development [18], which also played a key role in advancing a set of actionable indicators for assessing palliative care progress worldwide [12]. Among these indicators is one specific to children: the “number of hospices or palliative care services in the country with paediatric-specific palliative care programmes” [16]. For example, in Africa, the application of this indicator revealed a significant gap, with no known services or data for children’s palliative care in 32 out of 48 (67%) countries in the region [14].

The 2nd edition of the Global Atlas of palliative care [19] utilised data from 2017 [20,21] which included refinement of the original 4 categories to include 3a (isolated provision), 3b (generalised provision) and 4a (preliminary integration into mainstream, provision) and 4b (advanced integration). Within this 2nd edition of the Atlas, they reported on palliative care development generally using the revised categories but not specifically on children’s palliative care. Clelland et al. [22] conducted research on children’s palliative care by gathering data from in-country palliative care experts. They used a specific question within an online questionnaire designed to assess the overall level of palliative care development. Preliminary findings indicate that while the provision of children’s palliative care has grown and progressed, the highest level exists in only 21 countries, covering less than 10% of the global population under age 20. This care remains largely concentrated in high-income countries (HICs) despite the greatest need being in LMICs.

In 2020, Arias-Casis et al. published the first regional assessment of children’s palliative care development and provision in Europe, utilising data from the EAPC Atlas of palliative care [23]. These data included a single question regarding children’s palliative care service provision in the survey sent to the informants for the EAPC atlas, along with some follow-up questions to a specific group of children’s palliative care informants. They collected data from 51/54 (94%) European countries through the initial survey and 34/54 (62% countries) in follow-up discussions. Whilst noting the great developments in children’s palliative care provision within Europe, they recognised the disparities between high and low- and middle-income countries (LMICs).

Meanwhile, the International Children’s Palliative Care Network (ICPCN) has been mapping children’s palliative care since its inception in 2005, tracking global development [7,8], initially through word of mouth and updates from members and now through structured surveys to key children’s palliative care experts within countries. Thus, in 2023, the ICPCN conducted a comprehensive mapping exercise to assess the global status of children’s palliative care development in order to deepen the understanding of the current state of children’s palliative care worldwide and the developments across eight indicators. The insights gained will inform and support ongoing efforts in service development, advocacy, education, research, and access to essential medicines.

## 3. Method

A cross-sectional survey was undertaken to review the status of children’s palliative care around the world between June and December 2023. The survey was based on previous surveys undertaken by ICPCN, the levels of palliative care development [19], and the WHO Conceptual model for palliative care development [11] and consisted of 27 questions. Eight indicators for children’s palliative care development were included (Table 1), with participants being asked to estimate their perceived level of children’s palliative care development within their country, with the responses to other questions being used in triangulation of the data. Five levels were utilised in the survey (Table 2). These were aligned to the categories of palliative care development outlined in the Global Atlas of Palliative Care [19] and ICPCN’s previous mapping so that a comparison over time can be made (See Supplementary Materials).

Children’s palliative care experts and key stakeholders were purposively sampled and identified through previous mapping surveys, ICPCN’s membership and networks. The survey was distributed to participants from 167 countries out of a total of 197 countries (85%) (193 recognised by the United Nations, plus 4 additional non-UN recognised countries or states where ICPCN has long-standing contacts). In the remaining 30 countries (15%), we were unable to identify a key stakeholder in children’s palliative care. Participants were invited to complete the online survey within a five-week period, and the link to complete the survey via SurveyMonkey was sent to them via email. Where no response was received, several follow-up emails were sent. In countries where we had not received a response within 3 months, further contacts were identified, and the survey was sent to these new contacts. The survey was opened in August 2023 and closed after five months of follow-up for responses. Attempts were made to find experts in the countries where we had no contact through the regional palliative care associations. Data were confidential but not anonymous. All data was secured in a password-protected digital environment. The regional palliative care associations were also asked to validate the data (anonymised) for their region in order to identify any obvious discrepancies or misunderstandings regarding children’s palliative care development. Due to the type of data being collected, descriptive analysis was carried out. The survey has been reported in accordance with the Checklist for Reporting of Survey Studies (CROSS) [24] see Supplementary Materials.

## 4. Results

Data was collected for 131/167 countries (78% of countries with a known contact, 66% of total countries), with no responses from 36/167 countries (22% of surveys sent). The data for 123 countries was provided via the online survey; six were provided in a Word/PowerPoint format as they were unable to access the survey online, and two were partial responses by email that just contained the estimated level of children’s palliative care development for those countries. Duplicate responses were received from participants in five countries, including one respondent who completed it twice and several duplicates where the invited respondent had shared their link. If they did not agree with each other, further work was undertaken in order to reach a consensus. A total of 66% of the respondents were doctors and 15% nurses. Where there was no response from a country, or there was no known contact, that country was assigned to level 1 (recognising that no response or no known children’s palliative care expert does not necessarily mean that there is nothing going on in that country).

### 4.1. Indicator 1: Perceived Levels of Children’s Palliative Care Development

**Figure 2:**
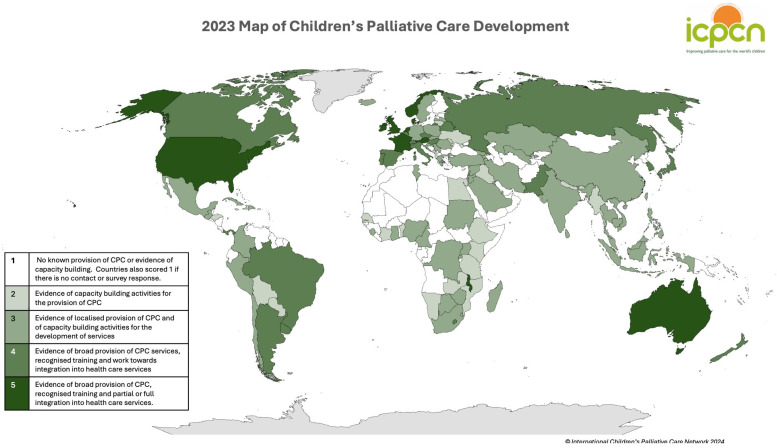
A map of the levels of children’s palliative (CPC) care development in 2023.

The levels of development in 2023 can be found in Table 3 and Figure 2, with 42% of countries still being at the lowest level of development and only 6% at the highest level. The data for each country is available at https://icpcn.org/mapping-cpc-development/ (accessed on 28 February 2025), and the list of countries and corresponding levels is available in the Supplementary Materials.

There were some significant movements between the different levels (Table 4)—in some countries (*n* = 59), there was an improvement with higher levels of children’s palliative care development. Meanwhile, in other countries (*n* = 17), it appeared that children’s palliative care had regressed, although in some cases, this was due to a lack of response rather than actual change in the level of care provision.

**Figure 3:**
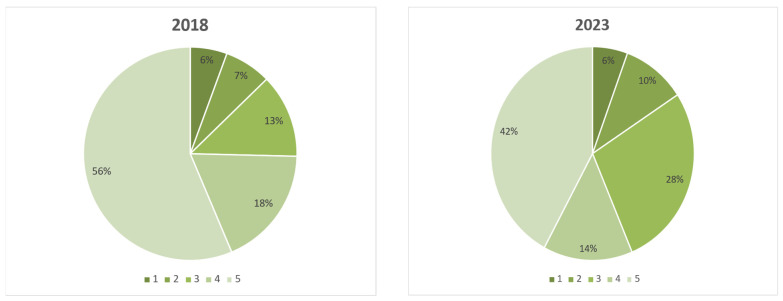
The percentage of countries at the different levels of children’s palliative care development in 2018 and 2023.

The overall net result of the movement showed an increase in children’s palliative care development globally, with fewer countries being at level 1 and an increase in countries at levels 3 and 4 (Figure 3). Out of the 11 countries identified as level 5 in 2018, 5 remained at level 5, 5 had dropped in level, and in one country, we were unable to obtain a response.

### 4.2. Indicator 2: Existence of National Organisations for Children’s Palliative Care

Twenty-seven countries reported that they had a national body for children’s palliative care, 72 said their national organisation included adults and children’s palliative care, and 70 said that they had a national professional association or special interest group for those working in children’s palliative care. Out of those reporting at least 1 national body/professional association, 8 countries reported having all three and 29 countries reported having two out of the three.

### 4.3. Indicator 3: Models of Children’s Palliative Care

Five different models of care were suggested, including the provision of children’s palliative care in a hospital, in the community, in a hospice, at the child’s school or other. Respondents were asked to describe all models that were available in their country across four different age groups (babies, children, adolescents, and adults, with some provision for children), with many countries having more than one model of care (Table 5).

### 4.4. Indicator 4: Inclusion of Children’s Palliative Care in National Policies

**Figure 4:**
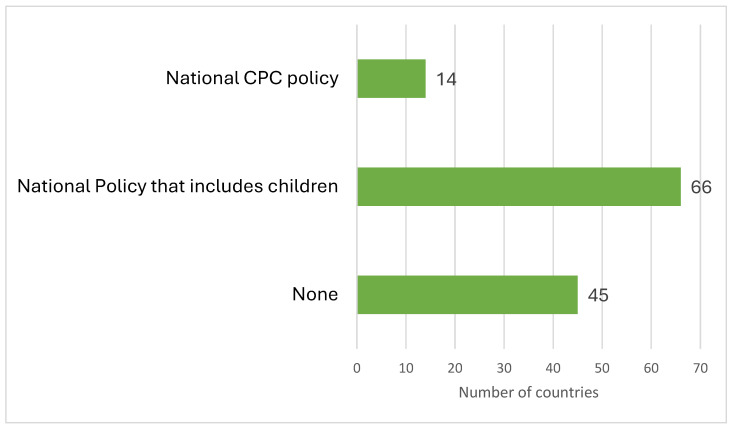
Number of countries with a policy related to children’s palliative care.

Of the 131 countries that responded, just 14 reported having dedicated national policies for children (Figure 4).

### 4.5. Indicator 5: Funding for Children’s Palliative Services

Respondents were asked how children’s palliative care services were funded in their country and whether they had secured national or local government funding. A total of 67 countries reported that they received some funding from their national government and 24 from their local government. A total of 33 countries reported that some of their children’s palliative care services received funding from grants and 57 that some services were funded through fundraising/philanthropy.

### 4.6. Indicator 6: Education for Children’s Palliative Care

The World Health Assembly (WHA) Resolution [25] defines three levels of training: “basic training and continuing education on palliative care; intermediate training for all routinely work with patients with life-threatening illnesses; and specialist palliative care training” [25]. Out of the 131 countries, 67 reported having basic training and continuing education on children’s palliative care available in their country, 40 had intermediate-level training for all routinely working with children with life-threatening illnesses, and 40 reported having specialist children’s palliative care training available.

**Figure 5:**
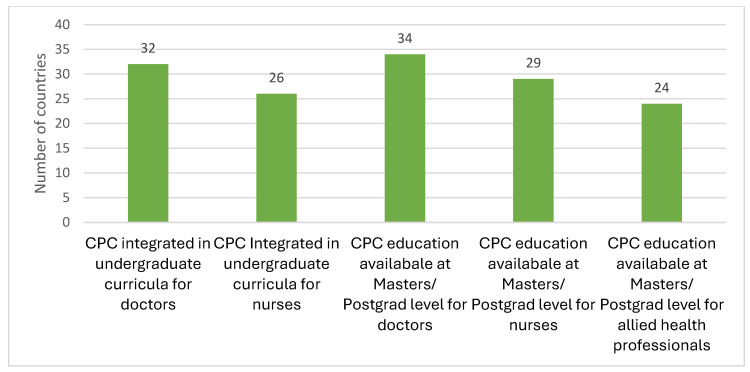
Availability of undergraduate and postgraduate education on children’s palliative care.

Respondents were also asked about the availability of undergraduate and postgraduate education in children’s palliative care for doctors, nurses and Allied Health Professionals (Figure 5).

### 4.7. Indicator 7: Access to Essential Medicines for Children’s Palliative Care

**Figure 6:**
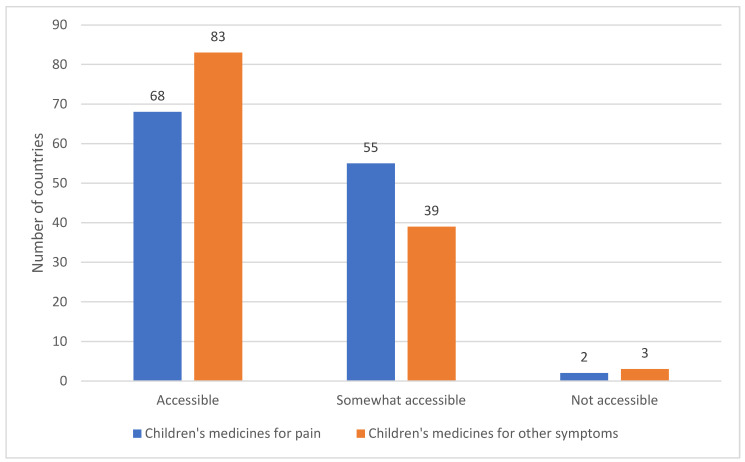
Availability of medicines for children’s palliative care.

The survey asked whether medicines for pain and for other symptoms in children’s palliative care were accessible, somewhat accessible (i.e., available < 50% of the time) or not accessible (Figure 6).

A total of 51 countries reported that their country’s essential medicine list did not include all of the essential medicines included in the WHO Essential Medicines List for children [26] needed for children’s palliative care.

### 4.8. Indicator 8: Research

The survey asked for contact details for researchers in their country who were leading research in children’s palliative care. A total of 79 countries provided information on leaders for children’s palliative care research in their country.

## 5. Discussion

Global progress in children’s palliative care is promising, but significant gaps remain in ensuring all children and families in need can access these essential services. While this paper reviews the overall development of children’s palliative care, certain indicators suggest higher levels of advancement. For instance, countries with established national bodies or professional associations dedicated to palliative care—particularly those with organisations focused specifically on children—demonstrate a stronger advocacy base for children’s palliative care. Additionally, the presence of national policies that explicitly address children’s palliative care, either within broader palliative care policies or as stand-alone guidelines, is a key factor in the embedding of children’s palliative care within the health system. Access to in-country education and training programmes further supports sustainable development, equipping healthcare providers to deliver quality palliative care to children. Countries which had earlier developed palliative care services for adults also act as catalysts for the development of children’s palliative care [21].

Significant disparities persist in children’s palliative care access and availability across regions (Table 6). These inequities are apparent both in comparisons between individual countries and across broader regions. For instance, in nations experiencing humanitarian crises or armed conflict, children’s palliative care provision often suffers setbacks, even with the dedicated efforts of in-country experts. Despite these challenges, expanding children’s palliative care access remains essential, especially in regions with the most unmet needs.

**Figure 7:**
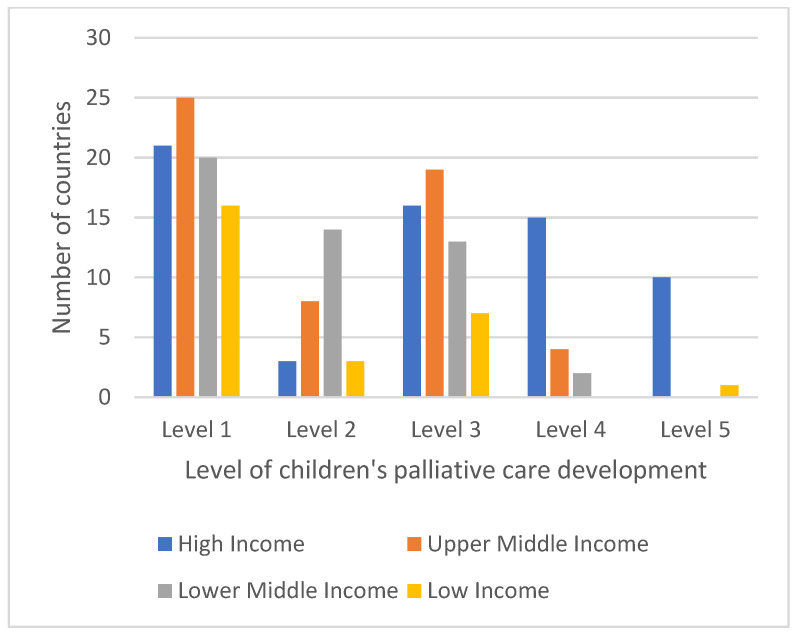
Levels of children’s palliative care development in 2023 across country income groups.

Four regions (AFRO, AMRO, EMRO and WPRO) have > 45% countries in level 1; in many of these countries, there was no known contact for children’s palliative care at the time of undertaking the survey, or there was no response, despite several approaches and changes in contact person. This is something that is reflected in the literature; for example, an agenda to develop children’s palliative care services in some of the EMRO countries was published in 2023 due to a need to develop services further within the region [27]. There are also clear differences in development depending on country income category (Figure 7), with 38% of high-income countries being in levels 4 and 5, whereas only 7% of upper-middle and 4% of lower-middle- and low-income countries were at the higher levels of children’s palliative care development, with no countries at the lower middle-income level and only one low-income country being at level 5.

Funding for children’s palliative care remains a challenge globally, and funding mechanisms for palliative care are not well understood [28]. Even in high-income countries, much of the funding is through charitable giving rather than government investment. Many funding streams have closed or reduced in recent years due to a lack of funding. This is even more of a challenge in LMICs. Indeed, a reduction in service provision in several countries across Africa can be linked to the closure or reduction of various funding programmes across the region.

Whilst the number of countries at level 5 remained the same (11), the perception was that some countries had progressed and others had regressed in level. Fluctuations in level of development, particularly at level 5, demonstrate that it can be hard to sustain children’s palliative care development in complex environments with competing funding demands, limited personnel and changing political environments. Sustaining some of the core elements at level 5, such as policy, funding, government support, and research, can be challenging.

More work is being undertaken to go deeper into the different indicators to complement this mapping, enhance the robustness and reliability of the findings, and examine the successes of countries that are continuing to develop services. For example:

- The availability of education in children’s palliative care at all three levels identified by the WHO in the World Health Assembly (WHA) Resolution [25] is essential for building capacity for children’s palliative care within a country [12]. The presence of palliative care, including children’s palliative care, in national curricula is a key indicator of sustainable and wide-reaching children’s palliative care skills and knowledge. Work is thus ongoing to ascertain the availability of education programmes on children’s palliative care globally, review their content and identify opportunities for collaboration and development.
- Access to medicines is another core component of the WHO conceptual model for palliative care [12]. Yet this study indicates the disparities in access to medicines, and it is anticipated that the lack of access to essential medicines for children’s palliative care is even greater as many of the countries that did not respond to the survey were LMICs with limited access to medicines. ICPCN, in conjunction with the St Jude Global Palliative Care program, is therefore looking deeper into the availability of different medications and formulations, along with the challenges of accessing them around the world.

To address the global disparities, increased efforts are required to identify and support champions of children’s palliative care, particularly in countries where local contacts are limited or nonexistent. Building the children’s palliative care leaders of the future is key, with a range of national, regional and international leadership programmes being delivered around the world, e.g., the Children’s Palliative Care Leadership Institute for South East Asia [29] or the Uganda Children’s Palliative Care Nurse Leadership Programme [30]. Strengthening our network of local advocates and expanding our database of contacts are key goals as we prepare to reassess children’s palliative care development in 2025. By broadening these connections, we aim to reduce the number of countries with limited engagement or response, helping to build a more comprehensive and accurate global picture of children’s palliative care access and provision. Utilisation of data such as the 2023 mapping data in advocacy campaigns is also important, an example of which was the 2024 #HatsOn4CPC campaign—a global awareness-raising campaign led by the ICPCN—which utilised stories and data to highlight some of the gaps in children’s palliative care data.

However, it is crucial to approach comparisons of this survey’s findings with caution. Differences in methodology and category definitions mean that the results of this mapping exercise may not align precisely with those of other surveys, such as the Global and Regional Atlases.

Significant work remains to ensure that all children globally have access to essential palliative care services. The recent World Innovation Summit of Health (WISH) report [31] recognised the limited progress that has been made in the development of palliative care for both adults and children since the World Health Assembly (WHA) Resolution on Palliative Care [25]. The report identified challenges and made recommendations for how we can respond and accelerate palliative care development [31]. The WHO conceptual model [12] offers a valuable framework for supporting, planning, and expanding children’s palliative care worldwide. This model emphasises key areas essential for comprehensive development: empowered communities, supportive policy frameworks, robust research, accessible education and training, availability of medicines, and integration of palliative care within health services. By focusing on these areas, we can work towards a holistic, child- and family-centred approach that addresses the diverse needs of children requiring palliative care.

## 6. Limitations

Mapping children’s palliative care globally presents numerous challenges, which introduce limitations to the findings of this study. One key challenge is identifying the most qualified individual to complete the mapping survey for each country, as responses often reflect the personal perspective of the respondent rather than the overall country perspective. Variations in perceived palliative care levels compared to previous years may result from different individuals completing the survey, as seen in instances where levels appeared lower than in the 2018 survey due to a shift in respondent perception. Furthermore, responses from representatives of smaller countries may capture a more comprehensive view than responses from larger countries, such as the United States or Russia, where the scope of palliative care services is broader and more complex. Additionally, given the nature of cross-sectional surveys, some data may be outdated by the time it is published, though regular mapping efforts allow us to build a picture of progress over time.

Challenges in past mappings of children’s palliative care have also included reliance on a single question to assess a country’s palliative care status, which was not always directed to a children’s palliative care expert. While some respondents may be knowledgeable about both adult and children’s palliative care in their country, it is essential to seek input directly from children’s palliative care specialists, which is the approach used for this survey. However, this approach has led to gaps in the data for countries where we lack contact with such experts or were unable to obtain a response. Regular engagement with local stakeholders and refining our approach to data collection, along with complementing expert-based assessments with more objective, data-driven evaluations where possible, are therefore crucial to building an accurate and comprehensive global picture of children’s palliative care.

## 7. Conclusions

The most recent mapping study has given us an opportunity to chart the progress of children’s palliative care development around the world. While there is still much progress to be made in ensuring all children have access to palliative care, examining the successes of countries that have made significant strides offers valuable insights. By identifying key factors that contributed to these advancements, we can support and guide other nations seeking to develop their own palliative care services for children and families. Recommendations for future work include ongoing mapping, refining the methods used, and supplementing this with more objective metrics to enhance the robustness of the findings. Learning from these leading examples provides a roadmap to promote sustainable growth in children’s palliative care globally, emphasising strategies that have proven effective and adaptable across various contexts. This collaborative approach not only encourages best practices but also strengthens the global commitment to equity in palliative care access for all children and their families.

## Figures and Tables

**Table 1:** Indicators covered within the Survey.

1.	Perceived levels of overall children’s palliative care development
2.	The existence of national associations for palliative care and children’s palliative care
3.	Availability of different service models for different age groups
4.	Inclusion of children’s palliative care in national policies
5.	Sustainable funding streams
6.	Education and capacity building
7.	Medicines availability
8.	Research capacity

**Table 2:** Levels of children’s palliative care development utilised in the study.

Level 1	No known provision of children’s palliative care or evidence of any capacity-building activities.
	*A country in this category shows no evidence of children’s palliative care services having been established and no preparatory work to develop such services. However, we acknowledge there may be instances where, despite our best efforts, current work has been unrecognized.* *This category also includes countries where we did not obtain a response -we acknowledge that a lack of response does not necessarily mean that there is nothing going on within a country. It also includes those countries with no known contact, which suggests that there is no or very little activity.*
Level 2	Evidence of capacity building activities for the provision of children’s palliative care.
	*A country in this category shows evidence of wide-ranging initiatives designed to create the organisational workforce and policy context for the development of palliative care services, although no service has been established yet. Developmental activities include attendance at, or organisation of, key conferences, personnel undertaking external training in palliative care, lobbying of policymakers and Ministries of Health and emerging plans for service development.*
Level 3	Evidence of localised provision of children’s palliative care and capacity-building activities for the development of services.
	*A country in this category shows evidence of children’s palliative care service delivery in individual hospital or community settings. Capacity-building activity includes service evaluation to build evidence of the need for sustainable funding for these services as well as activity to map the needs for further service development. Education and training initiatives are being provided in localities.*
Level 4	Evidence of multiple children’s palliative care services (broad provision), recognised training and work towards integration into health care services.
	*A country in this category will have many children’s palliative care services, but these may not yet be fully integrated into broader health services. There are good relationships with policymakers and funders, and children’s palliative care is becoming part of the national delivery and policy landscape. Education and training are of a high standard and widely available across the country.*
Level 5	Evidence of broad provision of children’s palliative care recognised training and partial or full integration into health care services.
	*A country in this category shows evidence of children’s palliative care services available in most areas. There are networks that aim to integrate and join up services and organisations/ professional bodies that advocate for the national development of children’s palliative care. Children’s palliative care is included in national and local health policy and planning. There is a coordinated approach to providing training and mandatory education on children’s palliative care, as well as a collaborative approach to developing research.*

**Table 3:** Levels of children’s palliative care development in 2023.

Level	Number of Countries	% of Countries
Level 1	83	42%
Level 2	28	14%
Level 3	55	28%
Level 4	20	10%
Level 5	11	6%
Total	197	100%

**Table 4:** Movement between levels since data collection in 2018.

Up 3 Levels	2
Up 2 levels	17
Up 1 level	40
Same	31
Down 1 level	14
Down 2 levels	1
Down 3 Levels	1
Down 4 levels	1

**Table 5:** Number of countries where the different models of children’s palliative care are available in at least one service.

	For Babies	For Children	For Adolescents /Young Adults	For Adults, but with Some Provision for Children
In the home	51	69	68	64
In the hospital	71	91	84	76
In a hospice	30	36	38	37
In the community	25	30	34	37
In schools	9	15	17	12

**Table 6:** Levels of children’s palliative care development in 2023 across WHO regions.

	AFRO	AMRO	EMRO	EURO	SEARO	WPRO
Level 1	23 (48%)	15 (45%)	10 (45%)	14 (26%)	4 (25%)	17 (65%)
Level 2	10 (22%)	5 (15%)	2 (9%)	7 (13%)	4 (25%)	0
Level 3	12 (26%)	7 (31%)	9 (41%)	16 (30%)	7 (44%)	4 (15%)
Level 4	1 (2%)	5 (15%)	1 (5%)	9 (17%)	0	4 (15%)
Level 5	1 (2%)	1 (3%)	0	7 (13%)	1 (6%)	1 (4%)
	46	33	22	53	16	26

## Data Availability

The data for each country is available at https://icpcn.org/mapping-cpc-development/ (accessed on 28 February 2025).

## Supplementary Materials

The following supporting information can be downloaded at: https://www.mdpi.com/article/10.3390/children12040440/s1, Table S1: Explanation of the Levels of CPC development in line with previous mapping and the Global Atlas. Table S2: Countries at the different levels of children’s palliative care development at the time of the 2023 survey. Table S3: Individual Country Levels For 2018 And 2023. Table S4: Checklist for Reporting of Survey Studies (CROSS) (Sharma et al. 2021 [24]).
