# Supplementary material for: Mapping of Children’s Palliative Care Development Globally in 2023
Source: Children (Basel). 2025 Mar 30;12(4):440. doi: 10.3390/children12040440 (PMC12025680; doi:10.3390/children12040440)
Supplement: Supplementary file 1 [file children-12-00440-s001.zip › children-3539108-supplementary.pdf]

SUPPLEMENTARY MATERIAL

Table S1. Explanation of the Levels of CPC development in line with previous mapping and the Global Atlas.

Mapping Survey 2019		Mapping Survey 2023			Global Atlas Level	
Original	Description	Original	Description	Revised	Original	Description
Group 1	**No known provision of children's palliative care**	Level 0	**No known provision of children's palliative care or evidence of any capacity building activities.** *A country in this category shows no evidence of children's palliative care services having been established and no preparatory work to develop such services. However, we acknowledge there may be instances where, despite our best efforts, current work has been unrecognised.*	Level 1	Group 1	**No known hospice-palliative care activity:** *Although we have been unable to identify any palliative care activity in this group of countries, we acknowledge there may be instances where, despite our best efforts, current work has been unrecognised.*
Group 2	**Evidence of capacity building activities for provision of children's palliative care**	Level 2	**Evidence of capacity building activities for the provision of children's palliative care.** *A country in this category shows evidence of wide-ranging initiatives designed to create the organisational, workforce, and policy context for the development of palliative care services, although no service has been established yet. Developmental activities include attendance at, or organisation of, key conferences, personnel undertaking external training in*	Level 2	Group 2	**Capacity building activity:** *In this group of countries, there is evidence of wide-ranging initiatives designed to create the organisational, workforce and policy capacity for hospice-palliative care services to develop, though no service has yet been established. The developmental activities include: attendance at, or organisation of, key conferences; personnel undertaking external training in palliative care; lobbying*

			palliative care, lobbying of policy makers and Ministries of Health and emerging plans for service development .			*of policy-makers and ministries of health; and incipient service development*
**Level 3**	**Evidence of localised provision of children's palliative care**	**Level 3**	**Evidence of localised provision of children's palliative care and of capacity building activities for the development of services.** <br>*A country in this category shows evidence of children's palliative care service delivery in individual hospital or community settings. Capacity-building activity includes service evaluation to build evidence of the need for sustainable funding for these services as well as activity to map the needs for further service development. Education and training initiatives are being provided in localities.*	**Level 3**	**Group 3**	**a) Isolated palliative care provision.** <br>*This group of countries is characterised by: the development of palliative care activism that is patchy in scope and not well supported; sourcing of funding that is often heavily donor- dependent; limited availability of morphine; and a small number of hospice-palliative care services that are often home-based in nature and relatively limited to the size of the population.* **b) Generalised palliative care provision.**

CONFIDENTIAL

							This group of countries is characterised by: the development of palliative care activism in a number of locations with the growth of local support in those areas; multiple sources of funding; the availability of morphine; a number of hospice-palliative care services from a community of providers that are independent of the healthcare system; and the provision of some training and education initiatives by the hospice organisations.
**Level 4**	**Evidence of board provision of children's palliative care, training and plans for development of services and integration into health care services**	Level 4	**Evidence of multiple children's palliative care services (broad provision), recognised training and work towards integration into health care services.**<br><br>*A country in this category will have many children's palliative care services but these may not yet be fully integrated into broader health services. There are good relationships with policy-makers and funders and children's palliative care is becoming part of the national delivery and policy landscape. Education and training is of a high standard and widely available across the country.*	Level 4		Group 4	**a) Countries where hospice-palliative care services are at a stage of preliminary integration into mainstream service provision.**<br><br>This group of countries is characterised by: the development of a critical mass of palliative care activism in a number of locations; a variety of palliative care providers and types of services; awareness of palliative care on the part of health professionals and local communities; the availability of morphine and some other strong pain-relieving medicines; limited impact of palliative care upon policy; the provision of a substantial number of training and education initiatives by a range of organisations; and interest in the

						concept of a national palliative care association.
Level 5	**Evidence of broad provision of children's palliative care, training and integration into health care services**	Level 5	**Evidence of broad provision of children's palliative care, recognised training and partial or full integration into health care services.**<br><br>*(A country in this category shows evidence of children's palliative care services available in most areas. There are networks that aim to integrate and join up services and organisations/ professional bodies that advocate for the national development of children's palliative care. Children's palliative care is included in national and local health policy and planning. There is a coordinated approach to providing training and mandatory education on children's palliative care as well as a collaborative approach to developing research).*	Level 5		**b) Countries where hospice-palliative care services are at a stage of advanced integration into mainstream service provision.**<br><br>*This group of countries is characterised by: the development of a critical mass of palliative care activism in a wide range of locations; comprehensive provision of all types of palliative care by multiple service providers; broad awareness of palliative care on the part of health professionals, local communities and society in general; unrestricted availability of morphine and all other strong pain-relieving medicines; substantial impact of palliative care upon policy, in particular upon public health policy; the development of recognised education centres; academic links forged with universities; and the*

CONFIDENTIAL

							existence of a national palliative care association.

CONFIDENTIAL

Table S2. Countries at the different levels of children's palliative care development at the time of the 2023 survey.

Level 5	Level 4	Level 3			Level 2
Australia<br>Austria<br>Denmark<br>France<br>Ireland<br>Malawi<br>Netherlands<br>Norway<br>Singapore<br>United Kingdom<br>United States of America	Argentina<br>Belarus<br>Belgium<br>Brazil<br>Canada<br>Czechia<br>Hong Kong<br>Hungary<br>Italy<br>Japan<br>Lesotho<br>New Zealand<br>Pakistan<br>Panama<br>Portugal<br>Russia<br>South Korea<br>Spain<br>Switzerland<br>Uruguay	Armenia<br>Botswana<br>Cambodia<br>Cameroon<br>Chile<br>China<br>Colombia<br>Democratic Republic of Congo<br>Croatia<br>Cuba<br>Dominican Republic<br>eSwatini<br>Georgia<br>Germany<br>Ghana<br>Greece<br>Guatemala	Iceland<br>India<br>Indonesia<br>Iran<br>Israel<br>Jordan<br>Kazakhstan<br>Kuwait<br>Latvia<br>Lebanon<br>Madagascar<br>Malaysia<br>Malta<br>Mexico<br>Nigeria<br>Peru<br>Philippines<br>Poland<br>Qatar	Romania<br>Rwanda<br>Saudi Arabia<br>Serbia<br>Sierra Leone<br>Slovakia<br>South Africa<br>Sri Lanka<br>Sudan<br>Sweden<br>Syria<br>Taiwan<br>Thailand<br>Tunisia<br>Turkey<br>Uganda<br>Uzbekistan<br>Vietnam<br>Zimbabwe	Albania<br>Andorra<br>Benin<br>Bhutan<br>Cote d'Ivoire<br>Egypt<br>Ethiopia<br>Gambia<br>Haiti<br>Honduras<br>Iraq<br>Jamaica<br>Kenya<br>Lithuania<br>Luxembourg<br>Moldova<br>Mozambique<br>Montenegro<br>Myanmar<br>Namibia<br>Nepal<br>Paraguay<br>Senegal<br>Tanzania<br>Timor-Leste<br>Ukraine<br>Zambia
**Level 1**					
Afghanistan*<br>Algeria<br>Angola<br>Antigua & Barbuda<br>Azerbaijan<br>Bahamas<br>Bahrain<br>Bangladesh<br>Barbados and eastern Caribbean<br>Belize<br>Bosnia and Herzegovina<br>Brunei<br>Bulgaria	Burkina Faso<br>Burundi<br>Cape Verde<br>Central African Republic<br>Chad<br>Comoros<br>Congo<br>Costa Rica<br>Cyprus<br>Djibouti<br>Dominica<br>Ecuador<br>El Salvador<br>Equatorial Guinea<br>Eritrea	Estonia<br>Fiji<br>Finland<br>Gabon<br>Grenada<br>Guinea<br>Guinea-Bissau<br>Guyana<br>Kiribati<br>Kosovo<br>Kyrgyzstan<br>Laos<br>Liberia<br>Libya<br>Liechtenstein<br>Maldives<br>Mali	Marshall Islands<br>Mauritania<br>Mauritius<br>Micronesia<br>Monaco<br>Mongolia<br>Morocco<br>Nauru<br>Nicaragua<br>Niger<br>North Korea<br>North Macedonia<br>Oman<br>Palau<br>Palestine	Papua New Guinea<br>Sait Kitts and Nevis<br>Saint Lucia<br>Saint Vincent and the Grenades<br>Samoa<br>San Marino<br>Sao Tome and Principle<br>Seychelles<br>Slovenia<br>Solomon Islands<br>Somalia<br>South Sudan	Suriname<br>Tajikistan<br>Togo<br>Tonga<br>Trinidad and Tobago<br>Turkmenistan<br>Tuvalu<br>United Arab Emirates<br>Vanuatu<br>Venezuela<br>Yemen

* denotes countries where there was no reply. [x] denotes countries where there was no contact

CONFIDENTIAL

Table S3. Individual Country Levels For 2018 And 2023.
Countries that are shaded grey are not currently recognised by the United Nations

Country	Contact/ Response 2023	WHO Region	Income Level as per 2023 (World Bank)	Level in 2023	Level in 20018	Movement
Afghanistan	No contact	Eastern Med	Low	1	1	=
Albania	Yes	Euro	Upper Middle	2	1	+1
Algeria	No contact	Africa	Lower Middle	1	1	=
Andorra	Yes	Europe	High	2	1	+1
Angola	No response	Africa	Lower Middle	1	1	=
Antigua and Barbuda	No contact	Western Pacific	High	1	1	=
Argentina	Yes	Americas	Upper Middle	4	3	+1
Armenia	Yes	Europe	Upper Middle	3	2	+1
Australia	Yes	Western Pacific	High	5	5	=
Austria	Yes	Europe	High	5	2	+3
Azerbaijan	No response	Europe	Upper Middle	1	1	=
Bahamas	Yes	Americas	High	1	1	=
Bahrain	No response	Eastern Med	High	1	1	=
Bangladesh	No response	South East Asia	Lower Middle	1	2	-1
Barbados	Yes	Americas	High	1	1	=
Belarus	Yes	Europe	Upper Middle	4	5	-1
Belgium	Yes	Europe	High	4	5	-1
Belize	No contact	Western Pacific	Upper Middle	1	1	=
Benin	Yes	Africa	Lower Middle	2	1	+1
Bhutan	Yes	South East Asia	Lower Middle	2	1	+1
Bolivia	Yes	Americas	Lower Middle	2	1	+1
Bosnia & Herzegovina	No response	Europe	Upper Middle	1	1	=
Botswana	Yes	Africa	Upper Middle	3	2	+1
Brazil	Yes	Americas	Upper Middle	4	2	+2
Brunei	No response	Western Pacific	High	1	1	=
Bulgaria	No contact	Europe	Upper Middle	1	1	=
Burkina Faso	Yes	Africa	Low	1	1	=
Burundi	No response	Africa	Low	1	1	=
Cambodia	Yes	Western Pacific	Lower Middle	3	1	+2
Cameroon	Yes	Africa	Lower Middle	3	2	+1
Canada	Yes	Americas	High	4	5	-1
Cape Verde	No contact	Africa	Lower Middle	1	1	=
Central African Republic	No contact	Africa	Low	1	1	=
Chad	No contact	Africa	Low	1	1	=
Chile	Yes	Americas	High	3	3	=
China	Yes	Western Pacific	Upper Middle	3	3	=
Colombia	Yes	Americas	Upper Middle	3	2	+1

CONFIDENTIAL

Comoros	No contact	Africa	Lower Middle	1	1	=
Congo (Republic of)	No contact	Africa	Low	1	1	=
Congo (DRC)	Yes	Africa	Low	3	2	+1
Costa Rica	No response	Americas	Upper Middle	1	5	-4
Cote D'Ivoire	Yes	Africa	Lower Middle	2	1	+1
Croatia	Yes	Europe	High	3	1	+2
Cuba	Yes	Americas	Upper Middle	3	1	+2
Cyprus	Yes	Europe	High	1	1	=
Czech Republic	Yes	Europe	High	4	3	+1
Denmark	Yes	Europe	High	5	5	=
Dijibouti	No response	Eastern Med	Lower Middle	1	1	=
Dominica	No contact	Americas	Upper Middle	1	1	=
Dominican Republic	Yes	Americas	Upper Middle	3	2	+1
Ecuador	No response	Americas	Upper Middle	1	2	-1
Egypt	Yes	Eastern Med	Lower Middle	2	2	=
El Salvador	No response	Americas	Upper Middle	1	1	=
Equatorial Guinea	No contact	Africa	Upper Middle	1	1	=
Eritrea	No contact	Africa	Low	1	1	=
Estonia	No response	Europe	High	1	1	=
Eswatini	Yes	Africa	Lower Middle	3	3	=
Ethiopia	Yes	Africa	Low	2	2	=
Fiji	No response	Western Pacific	Upper Middle	1	1	=
Finland	Yes	Europe	High	1	2	-1
France	Yes	Europe	High	5	4	+1
Gabon	No contact	Africa	Upper Middle	1	1	=
Gambia	Yes	Africa	Low	2	1	+1
Georgia	Yes	Europe	Upper Middle	3	3	=
Germany	Yes	Europe	High	3	5	-2
Ghana	Yes	Africa	Lower Middle	3	2	+1
Greece	Yes	Europe	High	3	3	=
Grenada	No contact	Americas	Upper Middle	1	1	=
Guatemala	Yes	Americas	Upper Middle	3	1	+2
Guinea	Yes	Africa	Lower Middle	1	1	=
Guinea-Bissau	No contact	Africa	Low	1	1	=
Guyana	Yes	Americas	High	1	1	=
Haiti	Yes	Americas	Lower Middle	2	1	+1
Honduras	Yes	Americas	Lower Middle	2	1	+1
Hong Kong	Yes	Western Pacific	High	4	3	+1
Hungary	Yes	Europe	High	4	3	+1
Iceland	Yes	Europe	High	3	1	+2
India	Yes	South East Asia	Lower Middle	3	4	-1

CONFIDENTIAL

Indonesia	Yes	South East Asia	Upper Middle	3	3	=
Iran	Yes	Eastern Med	Lower Middle	3	2	+1
Iraq	Yes	Eastern Med	Upper Middle	2	1	+1
Ireland	Yes	Europe	High	5	3	+2
Israel	Yes	Eastern Med	High	3	3	=
Italy	Yes	Europe	High	4	4	=
Jamaica	Yes	Americas	Upper Middle	2	1	+1
Japan	Yes	Western Pacific	High	4	2	+2
Jordan	Yes	Eastern Med	Lower Middle	3	2	+1
Kazakhstan	Yes	Europe	Upper Middle	3	1	+2
Kenya	Yes	Africa	Lower Middle	2	3	-1
Kiribati	No response	Western Pacific	Lower Middle	1	1	=
Kosovo	Yes	Europe	Upper Middle	1	1	=
Kuwait	Yes	Eastern Med	High	3	3	=
Kyrgyzstan	Yes	South East Asia	Lower Middle	1	2	-1
Laos	No contact	Western Pacific	Lower Middle	1	1	=
Latvia	Yes	Europe	High	3	4	-1
Lebanon	Yes	Eastern Med	Lower Middle	3	1	+2
Lesotho	Yes	Africa	Lower Middle	4	2	+2
Liberia	No response	Africa	Low	1	1	=
Libya	No response	Eastern Med	Upper Middle	1	1	=
Liechtenstein	No contact	Europe	High	1	1	=
Lithuania	Yes	Europe	High	2	1	+1
Luxembourg	Yes	Europe	High	2	1	+1
Madagascar	Yes	Africa	Low	3	1	+2
Malawi	Yes	Africa	Low	5	4	+1
Malaysia	Yes	Western Pacific	Upper Middle	3	3	=
Maldives	No contact	South East Asia	Upper Middle	1	1	=
Mali	No contact	Africa	Low	1	1	=
Malta	Yes	Europe	High	3	3	=
Marshall Islands	No contact	Western Pacific	Upper Middle	1	1	=
Mauretania	Yes	Africa	Lower Middle	1	1	=
Mauritius	Yes	Africa	Upper Middle	1	1	=
Mexico	Yes	Americas	Upper Middle	3	2	+1
Micronesia	No contact	Western Pacific	Lower Middle	1	1	=
Moldova	Yes	Europe	Upper Middle	2	2	=
Monaco	No contact	Europe	High	1	1	=
Mongolia	No response	Western Pacific	Lower Middle	1	1	=
Montenegro	Yes	Europe	Upper Middle	2	1	+1
Morocco	No response	Eastern Med	Lower Middle	1	1	=
Mozambique	Yes	Africa	Low	2	2	=

CONFIDENTIAL

Myanmar	Yes	South East Asia	Lower Middle	2	1	+1
Namibia	Yes	Africa	Upper Middle	2	2	=
Nauru	No contact	Western Pacific	High	1	1	=
Nepal	Yes	South East Asia	Lower Middle	2	1	+1
Netherlands	Yes	Europe	High	5	5	=
New Zealand	Yes	Western Pacific	High	4	5	+1
Nicaragua	No response	Americas	Lower Middle	1	1	=
Niger	No contact	Africa	Low	1	1	=
Nigeria	Yes	Africa	Lower Middle	3	2	+1
North Korea	No response	South East Asia	Low	1	1	=
North Macedonia	No response	Europe	Upper Middle	1	1	=
Norway	Yes	Europe	High	5	3	+2
Oman	No response	Eastern Med	High	1	1	=
Pakistan	Yes	Eastern Med	Lower Middle	4	2	+2
Palau	No contact	Western Pacific	Upper Middle	1	1	=
Palestine	Yes	Eastern Med	High	1	1	=
Panama	Yes	Americas	High	4	1	+3
Papua New Guinea	Yes	Western Pacific	Lower Middle	1	1	=
Paraguay	Yes	Americas	Upper Middle	2	1	+1
Peru	Yes	Americas	Upper Middle	3	1	+2
Philippines	Yes	Western Pacific	Upper Middle	3	2	+1
Poland	Yes	Europe	High	3	4	+1
Portugal	Yes	Europe	High	4	4	=
Qatar	Yes	South East Asia	High	3	1	+2
Romania	Yes	Europe	High	3	4	-1
Russia	Yes	Europe	Upper Middle	4	3	+1
Rwanda	Yes	Africa	Low	3	2	+1
Saint Kitts and Nevis	No response	Americas	High	1	1	=
Saint Lucia	No response	Americas	Upper Middle	1	1	=
Saint Vincent & Grenadines	No response	Americas	Upper Middle	1	1	=
Samoa	No response	Western Pacific	Lower Middle	1	1	=
San Marino	No contact	Europe	High	1	1	=
Sao Tome & Principe	No contact	Africa	Lower Middle	1	1	=
Saudi Arabia	Yes	Eastern Med	High	3	2	+1
Senegal	Yes	Africa	Lower Middle	2	1	+1
Serbia	Yes	Europe	Upper Middle	3	2	+1
Seychelles	Yes	Africa	High	1	1	=
Sierra Leone	Yes	Africa	Low	3	1	+2
Singapore	Yes	South East Asia	High	5	4	+1
Slovakia	Yes	Europe	High	3	1	+2
Slovenia	No response	Europe	High	1	1	=

CONFIDENTIAL

Solomon Islands	No response	Western Pacific	Lower Middle	1	1	=
Somalia	No response	Eastern Med	Low	1	1	=
South Africa	Yes	Africa	Upper Middle	3	4	-1
South Korea	Yes	Western Pacific	High	4	3	+1
South Sudan	Yes	Africa	Low	1	1	=
Spain	Yes	Europe	High	4	4	=
Sri Lanka	Yes	South East Asia	Lower Middle	3	1	+2
Sudan	Yes	Eastern Med	Low	3	2	+1
Suriname	No contact	Americas	Upper Middle	1	1	=
Sweden	Yes	Europe	High	3	3	=
Switzerland	Yes	Europe	High	4	4	=
Syria	Yes	Eastern Med	Low	3	1	+2
Taiwan	Yes	South East Asia	High	3	2	+1
Tajikistan	No response	Europe	Lower Middle	1	1	=
Tanzania	Yes	Africa	Lower Middle	2	3	-1
Thailand	Yes	South East Asia	Upper Middle	3	3	=
Timor-Leste	Yes	South East Asia	Lower Middle	2	1	+1
Togo	No response	Africa	Low	1	2	-1
Tonga	Yes	Western Pacific	Upper Middle	1	1	=
Trinidad & Tobago	Yes	Americas	High	1	1	=
Tunisia	Yes	Eastern Med	Lower Middle	3	1	+2
Turkey	Yes	Europe	Upper Middle	3	2	+1
Turkmenistan	No contact	Europe	Upper Middle	1	1	=
Tuvalu	No contact	Western Pacific	Upper Middle	1	1	=
Uganda	Yes	Africa	Low	3	4	-1
Ukraine	Yes	Europe	Lower Middle	2	3	-1
United Arab Emirates	No response	Eastern Med	High	1	3	-2
United Kingdom	Yes	Europe	High	5	5	=
United States of America	Yes	Americas	High	5	5	=
Uruguay	Yes	Americas	High	4	4	=
Uzbekistan	Yes	Europe	Lower Middle	3	1	=2
Vanuatu	Yes	Western Pacific	Lower Middle	1	1	=
Venezuela	No response	Americas	-	1	2	-1
Vietnam	Yes	South East Asia	Lower Middle	3	2	+1
Yemen	No response	Eastern Med	Low	1	1	=
Zambia	Yes	Africa	Lower Middle	2	2	=
Zimbabwe	Yes	Africa	Lower Middle	3	3	=

CONFIDENTIAL

Table S4. Checklist for Reporting Of Survey Studies (CROSS) (Sharma et al 2021 [24]).

Section/topic	Item	Item description	Reported on page #
**Title and abstract**			
Title and abstract	1a	State the word "survey" along with a commonly used term in title or abstract to introduce the study's design.	Mapping
	1b	Provide an informative summary in the abstract, covering background, objectives, methods, findings/results, interpretation/discussion, and conclusions.	3
**Introduction**			
Background	2	Provide a background about the rationale of study, what has been previously done, and why this survey is needed.	5-8
Purpose/aim	3	Identify specific purposes, aims, goals, or objectives of the study.	8
**Methods**			
Study design	4	Specify the study design in the methods section with a commonly used term (e.g., cross-sectional or longitudinal).	8-9
Data collection methods	5a	Describe the questionnaire (e.g., number of sections, number of questions, number and names of instruments used).	8-9
	5b	Describe all questionnaire instruments that were used in the survey to measure particular concepts. Report target population, reported validity and reliability information, scoring/classification procedure, and reference links (if any).	8-9
	5c	Provide information on pretesting of the questionnaire, if performed (in the article or in an online supplement). Report the method of pretesting, number of times questionnaire was pre-tested, number and demographics of participants used for pretesting, and the level of similarity of demographics between pre-testing participants and sample population.	N/A
	5d	Questionnaire if possible, should be fully provided (in the article, or as appendices or as an online supplement).	Supplementary material
Sample characteristics	6a	Describe the study population (i.e., background, locations, eligibility criteria for participant inclusion in survey, exclusion criteria).	9-10
	6b	Describe the sampling techniques used (e.g., single stage or multistage sampling, simple random sampling, stratified sampling, cluster sampling, convenience sampling). Specify the locations of sample participants whenever clustered sampling was applied.	9-10
	6c	Provide information on sample size, along with details of sample size calculation.	9-10
	6d	Describe how representative the sample is of the study population (or target population if possible), particularly for population-based surveys.	9-10
Survey administration	7a	Provide information on modes of questionnaire administration, including the type and number of contacts, the location where the survey was conducted (e.g., outpatient room or by use of online tools, such as SurveyMonkey).	9-10
	7b	Provide information of survey's time frame, such as periods of recruitment, exposure, and follow-up days.	9-10
	7c	Provide information on the entry process:<br>–>For non-web-based surveys, provide approaches to minimize human error in data entry.<br>–>For web-based surveys, provide approaches to prevent "multiple participation" of participants.	9-10

CONFIDENTIAL

Study preparation	8	Describe any preparation process before conducting the survey (e.g., interviewers' training process, advertising the survey).	9-10
Ethical considerations	9a	Provide information on ethical approval for the survey if obtained, including informed consent, institutional review board [IRB] approval, Helsinki declaration, and good clinical practice [GCP] declaration (as appropriate).	31
	9b	Provide information about survey anonymity and confidentiality and describe what mechanisms were used to protect unauthorized access.	8-9
Statistical analysis	10a	Describe statistical methods and analytical approach. Report the statistical software that was used for data analysis.	N/A
	10b	Report any modification of variables used in the analysis, along with reference (if available).	N/A
	10c	Report details about how missing data was handled. Include rate of missing items, missing data mechanism (i.e., missing completely at random [MCAR], missing at random [MAR] or missing not at random [MNAR]) and methods used to deal with missing data (e.g., multiple imputation).	10-11
	10d	State how non-response error was addressed.	N/A
	10e	For longitudinal surveys, state how loss to follow-up was addressed.	N/A
	10f	Indicate whether any methods such as weighting of items or propensity scores have been used to adjust for non-representativeness of the sample.	N/A
	10g	Describe any sensitivity analysis conducted.	N/A
**Results**			
Respondent characteristics	11a	Report numbers of individuals at each stage of the study. Consider using a flow diagram, if possible.	9-10
	11b	Provide reasons for non-participation at each stage, if possible.	N/A
	11c	Report response rate, present the definition of response rate or the formula used to calculate response rate.	9-10
	11d	Provide information to define how unique visitors are determined. Report number of unique visitors along with relevant proportions (e.g., view proportion, participation proportion, completion proportion).	N/A
Descriptive results	12	Provide characteristics of study participants, as well as information on potential confounders and assessed outcomes.	N/A
Main findings	13a	Give unadjusted estimates and, if applicable, confounder-adjusted estimates along with 95% confidence intervals and p-values.	N/A
	13b	For multivariable analysis, provide information on the model building process, model fit statistics, and model assumptions (as appropriate).	N/A
	13c	Provide details about any sensitivity analysis performed. If there are considerable amount of missing data, report sensitivity analyses comparing the results of complete cases with that of the imputed dataset (if possible).	N/A
**Discussion**			
Limitations	14	Discuss the limitations of the study, considering sources of potential biases and imprecisions, such as non-representativeness of sample, study design, important	16

CONFIDENTIAL

		uncontrolled confounders.	
Interpretations	15	Give a cautious overall interpretation of results, based on potential biases and imprecisions and suggest areas for future research.	10-15
Generalizability	16	Discuss the external validity of the results.	N/A
**Other sections**			
Role of funding source	17	State whether any funding organization has had any roles in the survey's design, implementation, and analysis.	32
Conflict of interest	18	Declare any potential conflict of interest.	31
Acknowledgements	19	Provide names of organizations/persons that are acknowledged along with their contribution to the research.	32

CONFIDENTIAL
